# Decision‐Making on First‐Line Treatment Alternatives in Chronic Lymphocytic Leukemia: A Multi‐Criteria Decision Analysis to Elicit Stakeholders' Preferences in Italy

**DOI:** 10.1002/hon.70274

**Published:** 2026-11-05

**Authors:** Paolo Sportoletti, Felice Bombaci, Chiara Cernetti, Alessandra Di Costanzo, Francesca Fiorentino, Sara Manurita, Andrea Marcellusi, Riccardo Mercati, Massimo Riccaboni, Chiara Vassallo, Antonio Cuneo

**Affiliations:** ^1^ Dipartmento di Medicina e Chirugia Centro di Ricerca Emato‐Oncologica (CREO) Universitá di Perugia Perugia Italy; ^2^ AIL Patient Groups Torino Italy; ^3^ BeOne Medicines Italy Srl Milan Italy; ^4^ IQVIA Solutions Italy Srl, Real World Solutions Milan Italy; ^5^ Department of Pharmaceutical Science University of Milan Milan Italy; ^6^ AxES IMT School for Advanced Studies Lucca Italy; ^7^ IUSS Pavia Italy; ^8^ Department of Medical Sciences University Hospital of Ferrara Ferrara Italy

**Keywords:** B‐cell lymphoma 2 inhibitors, chronic lymphocytic leukemia, first‐line therapy, Italy, multi‐criteria decision analysis, surveys and questionnaires, tyrosine kinase inhibitors

## Abstract

Chronic lymphocytic leukemia (CLL) is the most common form of leukemia in Western countries. The introduction of targeted therapies has expanded first‐line treatment options, which differ across multiple relevant dimensions, making therapeutic decisions increasingly challenging. A multi‐criteria decision analysis (MCDA) was conducted to identify and quantify the importance of key treatment selection criteria and to evaluate available first‐line alternatives for patients with mutated and unmutated immunoglobulin heavy‐chain variable region (IGHV^mut^/IGHV^unmut^) gene status, from the perspective of clinicians, patients, payers, and methodologists. The analysis focused on targeted therapies recommended by European Society for Medical Oncology (ESMO) guidelines and reimbursed in Italy, including ibrutinib, acalabrutinib, zanubrutinib, venetoclax + ibrutinib, and venetoclax+obinutuzumab. The MCDA was developed in accordance with International Society for Pharmacoeconomics and Outcomes Research (ISPOR) Good Practice recommendations, using the MACBETH (Measuring Attractiveness by a Categorical‐Based Evaluation Technique) method to elicit stakeholder preferences. Decision criteria were selected based on literature review, stakeholder input, and data availability. Preferences were collected from 20 stakeholders (nine clinicians, five patients, six payers/methodologists) via structured questionnaires. Six criteria were selected: efficacy, safety, quality of life (QoL), drug cost, convenience of administration, and treatment duration. Efficacy and safety emerged among the most important criteria across all groups, with efficacy particularly prioritized in the IGHV^unmut^ population. Among the other criteria, notable differences emerged between stakeholder groups: clinicians assigned relatively greater importance to treatment duration, especially in the IGHV^mut^ population; patients prioritized QoL more than other groups; payers and methodologists gave more weight to drug cost than clinicians and patients. In the IGHV^mut^ population, venetoclax + ibrutinib, and zanubrutinib were the most valued options. In the IGHV^unmut^ population, second‐generation Bruton tyrosine kinase inhibitors (zanubrutinib and acalabrutinib) ranked highest. Sensitivity analyses confirmed the robustness of these findings. This is the first MCDA study applied to CLL treatment in Italy. It highlights the value of structured, stakeholder‐informed approaches in supporting value‐based treatment decisions.

## 1 Introduction

Chronic lymphocytic leukemia (CLL) represents the most prevalent form of leukemia in Western countries, accounting for approximately 30% of all leukemia cases, with approximately 2600 new diagnoses estimated in Italy in 2024 [1, 2]. CLL is characterized by a highly variable clinical course, ranging from indolent forms that remain asymptomatic for years to aggressive subtypes requiring early intervention [3]. According to guidelines, treatment should be initiated only when the disease is in advanced or symptomatic stage [4]. In such cases, treatment decisions should consider both expected clinical course based on prognostic biomarkers and patient‐specific aspects such as comorbidities, concurrent medications, and individual preferences [1, 4, 5]. The established factors guiding therapeutic choices in the first‐line setting include the mutational status of the immunoglobulin heavy‐chain variable region (IGHV) gene, the patient age, clinical fitness, and *TP53* mutational status [1, 4]. Indeed, current European Society for Medical Oncology (ESMO) first‐line treatment algorithms [4] stratify patients according to IGHV status and *TP53* aberrations. Because *TP53* aberrations are observed in only a minority of previously untreated patients [6], IGHV mutational status represents one of the main drivers of treatment selection in routine clinical practice, with patients with unmutated IGHV (IGHV^unmut^) experiencing a more aggressive disease course, shorter remissions, and poorer survival outcomes than those with mutated IGHV (IGHV^mut^) [4, 6].

In recent years, the clinical first‐line management of CLL has shifted from chemotherapy‐based regimens to targeted therapies, including Bruton tyrosine kinase inhibitors (BTKis) (ibrutinib, acalabrutinib, zanubrutinib) and the B‐cell lymphoma 2 antagonist venetoclax [3, 4]. This transition is reflected in current treatment guidelines [4] and supported by multiple Phase III trials on patients with previously untreated CLL [7, 8, 9, 10, 11, 12].

As a result, the selection of first‐line therapy for CLL has become increasingly complex due to the wide range of available options, which differ significantly in terms of efficacy across patient profiles, mechanisms of action, administration routes, treatment duration, safety profiles, and cost implications, making direct comparisons challenging [4, 13]. This diversity, combined with the variability in patient characteristics and prognostic factors—such as IGHV mutation—and the limited availability of structured evidence on stakeholder preferences, highlights the need for a multi‐dimensional and multi‐stakeholder approach to therapeutic decision‐making [14, 15]. Moreover, in this framework, the integration of patient perspectives is increasingly advocated, as emphasized by the European Joint Clinical Assessment, which recognizes patients as active contributors to decision‐making [16]. The objective of this study is to identify and quantify the importance of key treatment selection criteria and to assess the value of first‐line targeted therapies for CLL in Italy for patients with either IGHV^mut^ or IGHV^unmut^ status. The evaluation is carried out through a multi‐criteria decision analysis (MCDA) that integrates the perspectives of patients, clinicians, and payers.

## 2 Methods

All study phases were conducted coherently with guidelines from the International Society for Pharmacoeconomics and Outcomes Research (ISPOR) MCDA Emerging Good Practices Task Force [17, 18]. This study employed MACBETH (Measuring Attractiveness by a Categorical‐Based Evaluation Technique) as the preference elicitation method [19] due to its ability to elicit stakeholders' preferences while requiring respondents to provide only qualitative and non‐numerical judgments [20, 21, 22, 23, 24].

First, relevant therapeutic alternatives and stakeholders were identified. The study included all first‐line targeted therapies recommended by ESMO guidelines [4] for patients with IGHV^mut^ or IGHV^unmut^ CLL that were reimbursed in Italy at the time of the study [25, 26, 27, 28], which include both continuous treatments with BTKis and fixed‐duration therapies with venetoclax. Although potentially suitable for patients with favorable genetic profiles, chemo‐immunotherapy was not considered, as targeted therapies should be prioritized due to superior survival outcomes [4]. Thus, selected alternatives for both IGHV^mut^ and IGHV^unmut^ patients were acalabrutinib, ibrutinib, venetoclax + ibrutinib, venetoclax + obinutuzumab, and zanubrutinib. The stakeholders included in the analysis were clinicians treating patients with CLL, patients receiving treatment, and methodologists and payers involved in reimbursement decisions. A core group of key opinion leaders (KOLs) comprising two hematologists, two methodologists, and one patient representative was convened to participate in two workshops: the first aimed at validating the decision criteria, and the second focused on reviewing and interpreting the results. In addition to this core group, 15 further stakeholders were invited to complete the questionnaire, resulting in a total of 20 respondents (nine hematologists, six methodologists/payers, one patient representative, and four additional patients). Hematologists were identified based on their recognized expertise in CLL management and scientific and academic standing, while ensuring balanced geographical representation across Northern, Central, and Southern Italy. Methodologists and payers were selected based on their expertise in pharmaceutical regulation, health technology assessment (HTA) processes, or healthcare‐related statistical methodologies. The patient representative was affiliated with AIL (Associazione Italiana contro le leucemie, linfomi e mieloma); the questionnaire for the additional patients was disseminated through the representative's patient network, and their identities remained anonymous to the study team.

Subsequently, relevant decision criteria were selected. Potential criteria were initially identified through a targeted literature review, searching for MCDA studies in onco‐hematology, rare diseases and orphan drugs, and Discrete Choice Experiments (DCEs) in CLL. Publications on rare diseases and orphan drugs were deemed relevant, given that CLL has a prevalence near the European Union threshold for rare diseases [29, 30], and orphan drugs are specifically intended for conditions falling under this classification [31]. DCEs were included due to their extensive use in the preference elicitation literature [32] and their suitability as a method for eliciting preferences within the MCDA framework [18]. Thirty‐four relevant publications were identified through searches on PubMed, cross‐references, and targeted research. A preliminary list of criteria was established based on those used in ≥ 15% of the reviewed articles, resulting in a total of 23 criteria. Next, these were reduced to nine upon verifying their compliance with ISPOR properties (i.e., completeness, non‐redundancy, non‐overlap, and preference independence) [18]. On July 12, 2024, the restricted panel of five representative stakeholders discussed and validated the criteria for inclusion in the final list, along with their operative definitions. Stakeholders were given the possibility to differentiate selected criteria between the IGHV^mut^ and IGHV^unmut^ populations.

Further literature research was then conducted to inform two performance matrices, one for the population with IGHV^mut^ CLL and one for the population with IGHV^unmut^ CLL, reporting the level of each alternative and in each criterion. In situations where data were not available for all the therapeutic alternatives, hypothetical levels based on existing literature were assessed. Finally, differences between the best and worst performance levels in each criterion (i.e., improvements) were calculated [23].

A questionnaire was developed in two versions: one for clinicians, payers, methodologists, and the patient representative, and one with simplified clinical wording for patients. Before accessing the questionnaire, participants were presented with an introductory page outlining the study objectives and an informed consent page describing key aspects of participation, including guarantees of anonymity; access was granted only upon consent. The questionnaire was divided into two main sections, according to the MACBETH method (details in Appendix). The first section, *Weighting*, led each stakeholder to implicitly assign a weight, or relative importance, from 0% to 100% to all criteria, with higher weights indicating more significant criteria improvements. The second section, *Scoring*, aimed to elicit stakeholders' preferences for changes in performance within each criterion. Stakeholders' answers from the *Scoring* section were turned into numerical scores through the MACBETH optimization algorithm [33] and represented as individual value functions, which could be convex, concave, or linear.

Based on weighting and scoring of each respondent, individual aggregate scores for each therapeutic alternative were computed by applying an additive aggregation model [24, 34]. Aggregate scores could range from 0 to 100 and represented the overall values of alternatives as per stakeholders' preferences. Mean aggregate scores were calculated to rank the included alternatives, both within each stakeholder group (i.e., clinicians, methodologists/payers, and patients) and overall, based on the average of group‐level aggregate scores.

Questionnaire answers were collected between November 7, 2024, and January 15, 2025. On February 18, 2025, the second workshop was held to review the results and discuss potential sensitivity analyses, which were subsequently conducted by varying the parameters considered most uncertain.

## 3 Results

### 3.1 Criteria: Identifying Key Decision Factors

As preliminarily described in a previously published work [35], our MCDA included six criteria, for both IGHV^mut^ and IGHV^unmut^ populations.

1. Efficacy: Progression‐free survival (PFS) rates at 42 months (most updated comparable follow‐up) from baseline [36, 37, 38, 39, 40].
2. Safety: Rates of treatment discontinuation due to adverse events (AEs) at follow‐up ranging from 26 to 29 months (most updated comparable follow‐up) [7, 10, 11, 12, 41].
3. Quality of life (QoL): Whether a statistically significant improvement in Global Health Status (GHS) score at 6 months from baseline was demonstrated or not [39, 42, 43, 44].
4. Drug cost: Average annual acquisition cost over the first 3 years of treatment, weighted by PFS at each year. Specifically, the acquisition costs for years 1, 2, and 3 were multiplied by the corresponding yearly PFS rates to reflect treatment discontinuation due to disease progression or death. These yearly costs were then averaged to obtain a PFS‐adjusted annual cost estimate. Acquisition costs were valued net of approximated overall discount, including discount negotiated with Agenzia Italiana del Farmaco (AIFA), tender, and commercial discount (IQVIA statistical elaboration on expenditure data from a hospital panel) [25, 26, 27, 28, 36, 37, 38, 39, 40, 45, 46, 47, 48, 49, 50].
5. Convenience of administration: Composite of administration mode (oral or intravenous, for each drug included in the therapy), administration setting (home or hospital, for each drug included in the therapy), hospital accesses due to administration, and dosing schedule (fixed or variable dosage) [46, 47, 48, 49, 50].
6. Treatment duration: Fixed or until progression [46, 47, 48, 49, 50].

Selection of PFS measurements specific for IGHV^mut^ and IGHV^unmut^ populations led to performance matrices differentiation across the two mutations. Since no QoL data consistent with the selected criterion were available for acalabrutinib, its performance was assumed equal to the other BTKis (ibrutinib and zanubrutinib). The detailed performance matrix is available in Table 1.

**TABLE 1:** Performance matrix of the alternatives.

Criterion	Description	ACA	IBR	VEN + IBR	VEN + OBI	ZAN	Source
Efficacy	PFS rate at 42 months						
	IGHV^mut^	81.0%	83.7%	90.0%	85.2%	85.4%	[36, 37, 38, 39, 40]
	IGHV^unmut^	81.9%	78.2%	69.8%	77.5%	80.9%	[36, 37, 38, 39, 40]
Safety	Treatment discontinuation due to AEs (26–29 months' follow‐up)	8.9%	12.1%	10.4%	16.0%	8.3%	[7, 10, 11, 12, 41]
Quality of life	Whether a statistically significant improvement in GHS at 6 months from baseline was demonstrated	Yes^a^	Yes	No	Yes	Yes	[39, 42, 43, 44]
Drug cost	Annual average acquisition cost of the first 3 years of treatment weighted by PFS, net of approximated overall discount^a^	€40,000	€37,000	€28,000	€17,000	€34,000	[25, 26, 27, 28, 36, 37, 38, 39, 40, 45, 46, 47, 48, 49, 50]
Convenience of administration	Administration mode, administration setting, hospital accesses due to administration, constant/variable dosage	Oral, home, no accesses, constant dosage	Oral, home, no accesses, constant dosage	Oral + oral, hospital + home, 8 accesses, variable dosage	IV + oral, hospital + home, 15 accesses, variable dosage	Oral, home, no accesses, constant dosage	[46, 47, 48, 49, 50]
Treatment duration	Fixed duration or until progression	Until progression	Until progression	Fixed duration	Fixed duration	Until progression	[46, 47, 48, 49, 50]

Abbreviations: ACA = acalabrutinib; AE = adverse event; AIFA = Agenzia Italiana del Farmaco; GHS = Global Health Status; IBR = ibrutinib; IGHV^mut^ = immunoglobulin heavy‐chain variable mutated; IGHV^unmut^ = immunoglobulin heavy‐chain variable unmutated; IV = intravenous; MAT = moving annual total; OBI = obinutuzumab; PFS = progression‐free survival; VEN = venetoclax; ZAN = zanubrutinib.

^a^ Including discount negotiated with AIFA, tender, and commercial discount (IQVIA statistical elaboration on expenditure data from a hospital panel).

### 3.2 Weighting: Assessing the Relative Importance of Criteria

Figure 1 provides a breakdown of mean weights by stakeholder group and the average across the group‐specific means. Efficacy and safety emerged among the most important criteria across all stakeholder categories in both the IGHV^mut^ and IGHV^unmut^ populations. However, efficacy became even more prominent in the IGHV^unmut^ population, with an overall average of 31% compared with 22% in IGHV^mut^. This increase was particularly marked among clinicians, who assigned 36% weight to efficacy in the IGHV^unmut^ versus 21% in IGHV^mut^. While efficacy and safety were key priorities for both clinicians and patients, clinicians also attributed considerable importance to treatment duration, especially in the IGHV^mut^ population (17% in IGHV^unmut^ vs. 24% in IGHV^mut^), whereas patients placed relatively more weight on QoL (22% in both populations). Payers and methodologists similarly valued efficacy and safety but assigned greater relative importance to drug cost (14% in IGHV^unmut^ vs. 18% IGHV^mut^) than the other stakeholder groups, for whom this criterion consistently received the lowest weight. Convenience of administration, instead, showed moderate variation across groups and between the two populations.

**FIGURE 1:**
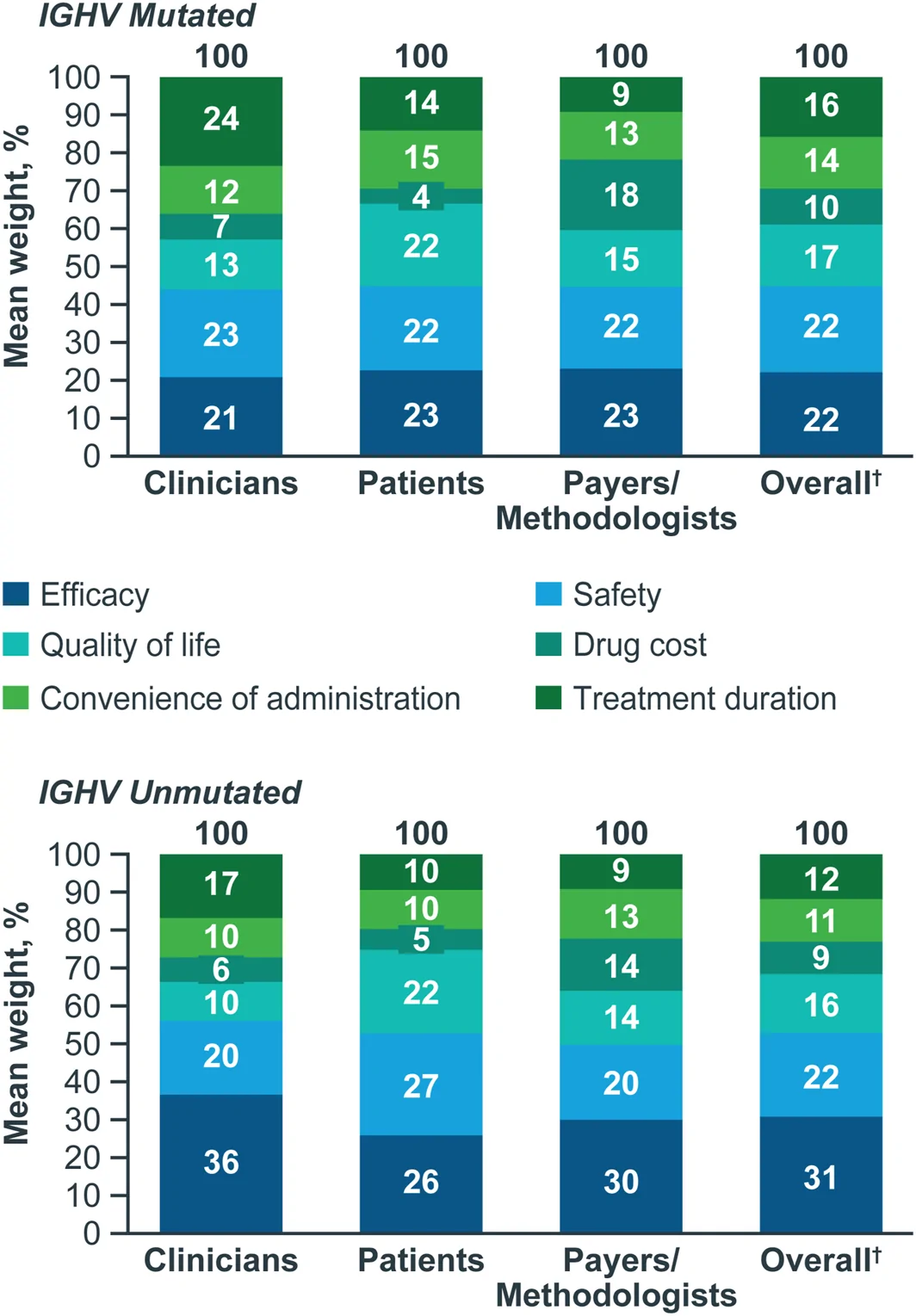
Mean weights by stakeholder group and overall, for each population. ^†^Calculated as the average of mean weights across stakeholder groups. Efficacy was [69.8%–81.9%]; quality of life was [Demonstrated improvement—Not demonstrated improvement]; convenience of administration was [oral, home, 0 accesses, constant dosage – IV + oral, 15 accesses hospital + home, variable dosage]; safety was [8.9%–16.0%]; drug cost was [€17,000–€40,000]; treatment duration was [Fixed—Until progression]. IGHV = immunoglobulin heavy‐chain variable; IV = intravenous.

### 3.3 Scoring: Assessing Preferences Within Criteria

Figure 2 illustrates the distribution of value function profiles across criteria. Overall, most respondents exhibited linear preferences across all criteria, indicating that they assigned equal value to improvements from the worst to intermediate level and from the intermediate to the best level of the criterion. Notably, the safety criterion showed the highest prevalence of non‐linear preferences (45%).

**FIGURE 2:**
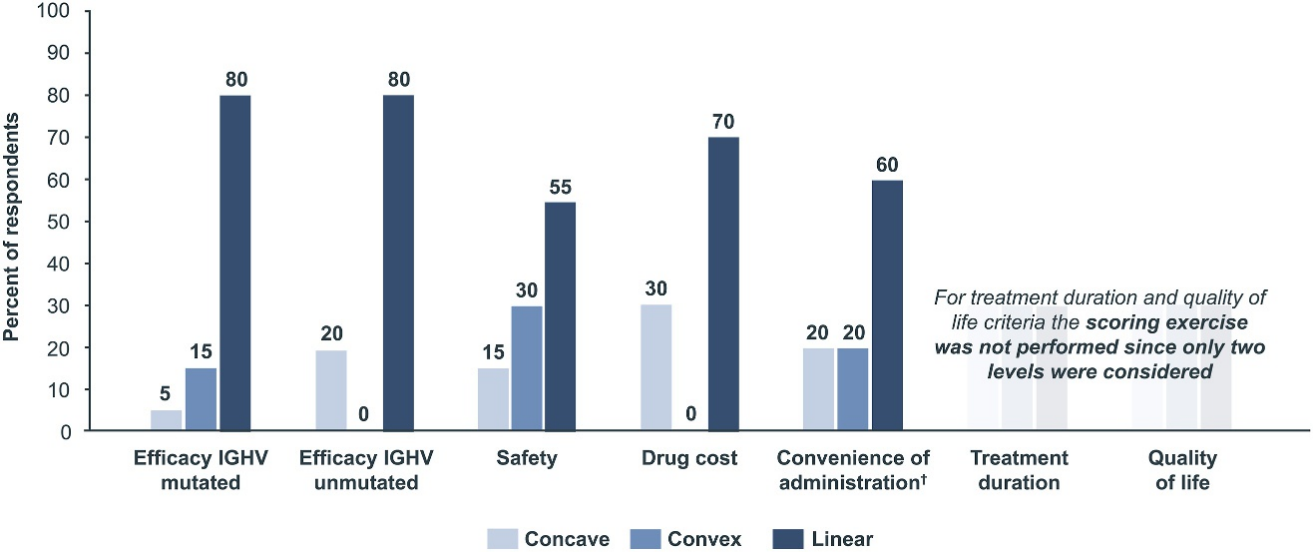
Proportion of value function profiles for each criterion in the sample. ^†^As for the convenience of administration, preferences are considered “linear” if respondents gave the same value to the change from intravenous + oral to oral + oral and to the change from oral + oral to oral. Preferences are considered “concave” if the change from intravenous + oral to oral + oral was considered more valuable than the change from oral + oral to oral. Preferences are considered “convex” if the change from intravenous + oral to oral + oral was considered less valuable than the change from oral + oral to oral. IGHV = immunoglobulin heavy‐chain variable.

### 3.4 Aggregate Scores: Assessing the Value of Considered Therapies

In the IGHV^mut^ population, as reported in Table 2 and Figure 3, venetoclax + ibrutinib (66.0) and zanubrutinib (65.3) achieved the highest overall aggregate score, calculated as the average across the group‐specific means, significantly outperforming the remaining treatment options. Although these therapies were consistently the most favored across all stakeholder groups, some differences in preference emerged: clinicians leaned toward venetoclax + ibrutinib, patients toward zanubrutinib, and payers rated both similarly. Other treatments had lower aggregate scores.

**TABLE 2:** Aggregate score and ranking by stakeholder group and overall for IGHV^mut^ population.

Therapy	Clinicians		Patients		Payers/methodologists		Overall^a^	
	Score	Ranking	Score	Ranking	Score	Ranking	Score	Ranking
Acalabrutinib	46.9	4th	57.6	3rd	48.0	4th	50.8	4th
Ibrutinib	44.5	5th	55.0	4th	47.9	5th	49.1	5th
Venetoclax + ibrutinib	72.0	1st	62.5	2nd	63.6	2nd	66.0	1st
Venetoclax + obinutuzumab	53.3	3rd	49.2	5th	52.4	3rd	51.6	3rd
Zanubrutinib	60.7	2nd	70.0	1st	65.2	1st	65.3	2nd

Abbreviation: IGHV^mut^ = immunoglobulin heavy‐chain variable mutated.

^a^ Calculated as the average of mean aggregate scores across stakeholder groups.

**FIGURE 3:**
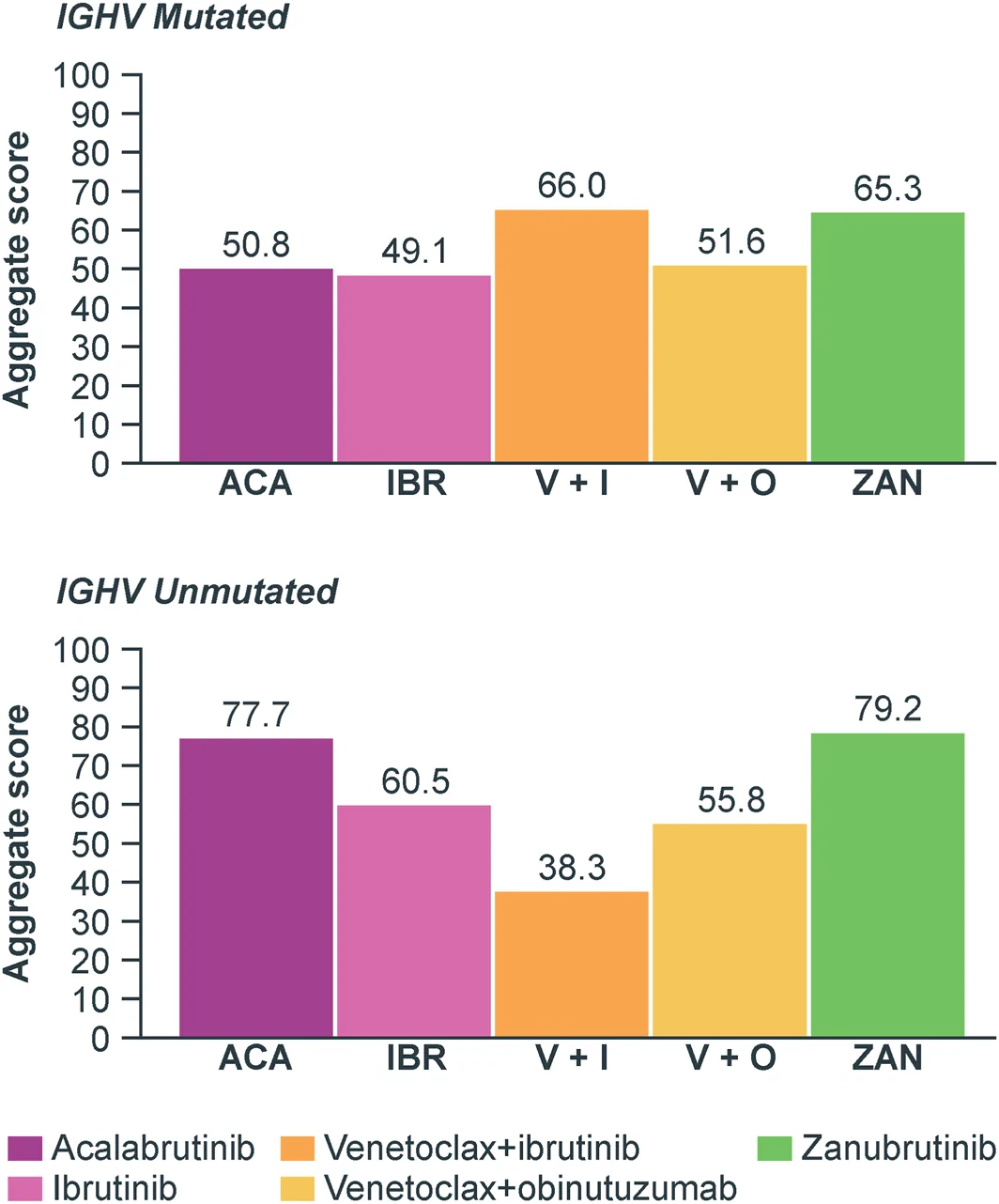
Overall^†^ aggregate scores by population. ^†^Calculated as the average of mean aggregate scores across stakeholder groups. ACA = acalabrutinib; IBR = ibrutinib; IGHV = immunoglobulin heavy‐chain variable; V + I = venetoclax + ibrutinib; V + O = venetoclax + obinutuzumab; ZAN = zanubrutinib.

In the IGHV^unmut^ population, as illustrated in Table 3 and Figure 3, the second‐generation BTKis zanubrutinib (79.2) and acalabrutinib (77.7) received the highest overall aggregate scores, followed by ibrutinib, venetoclax + obinutuzumab, and venetoclax + ibrutinib. Zanubrutinib and acalabrutinib consistently ranked highest across all stakeholder groups, with slight variations observed in the ranking of the remaining therapies.

**TABLE 3:** Aggregate score and ranking by stakeholder group and overall for IGHV^unmut^ population.

Therapy	Clinicians		Patients		Payers/methodologists		Overall^a^	
	Score	Ranking	Score	Ranking	Score	Ranking	Score	Ranking
Acalabrutinib	75.1	2nd	82.7	2nd	75.3	2nd	77.7	2nd
Ibrutinib	57.0	4th	64.7	3rd	59.8	3rd	60.5	3rd
Venetoclax + ibrutinib	40.5	5th	37.2	5th	37.1	5th	38.3	5th
Venetoclax + obinutuzumab	57.1	3rd	53.8	4th	56.4	4th	55.8	4th
Zanubrutinib	75.4	1st	84.1	1st	78.0	1st	79.2	1st

Abbreviation: IGHV^unmut^ = immunoglobulin heavy‐chain variable unmutated.

^a^ Calculated as the average of mean aggregate scores across stakeholder groups.

### 3.5 Sensitivity Analyses: Dealing With Uncertainty

To evaluate the robustness of the results, sensitivity analyses were conducted by varying parameters considered to be more uncertain.

1. Efficacy: In the absence of direct or indirect comparisons, efficacy values from randomized controlled trials were individually varied by ± 5% to address potential comparability issues.
2. Safety: Since at the considered safety follow‐up fixed‐duration treatments had ended while continuous ones were ongoing, higher discontinuation rates (worst safety level in the questionnaire) were applied to the latter to address this difference.
3. QoL: Since the criterion was defined as binary due to the lack of comparable quantitative data, and data were missing for one alternative, it was excluded in a sensitivity analysis.
4. Preference weights: To account for uncertainty in preferences, ± 20% variations were applied to weights of key criteria: efficacy and safety for all groups, and QoL, treatment duration, and drug cost for patients, clinicians, and payers, respectively.

By considering the overall aggregate score, for the IGHV^mut^ population, variations in efficacy levels and preference weights did not alter the main conclusions: venetoclax + ibrutinib and zanubrutinib consistently achieved the highest overall aggregate scores. When the QoL criterion was excluded, venetoclax + ibrutinib emerged as the top‐ranked option, followed by zanubrutinib. Furthermore, under the scenario assuming higher discontinuation rates for continuous treatments, fixed‐duration regimens (venetoclax + ibrutinib and venetoclax + obinutuzumab) showed the highest overall aggregate scores. For the IGHV^unmut^ population, all sensitivity analyses confirmed baseline results. Second‐generation BTKis (zanubrutinib and acalabrutinib) consistently ranked highest across all tested scenarios, with venetoclax + obinutuzumab approaching their overall aggregate scores in the safety sensitivity analysis.

## 4 Discussion

The first‐line treatment landscape for CLL, both IGHV^mut^ and IGHV^unmut^, has evolved with the introduction of novel targeted therapies, which differ across key aspects. In this context, a structured, multi‐dimensional approach is essential to guide informed decisions. This study is the first to apply MCDA to systematically evaluate available first‐line treatment options for CLL from diverse stakeholder perspectives. Results showed that efficacy and safety were consistently among the top priorities across stakeholder groups, though preferences varied by stakeholder type and IGHV status. As a result, venetoclax + ibrutinib and zanubrutinib were most favored in the IGHV^mut^ population, while zanubrutinib and acalabrutinib ranked highest in the IGHV^unmut^ population.

The adherence to ISPOR MCDA Good Practices [17, 18] and the use of a structured preference elicitation method ensured methodological rigor and transparency, enabling a robust and quantitative comparison of therapies and the identification of the trade‐offs that stakeholders are willing to make.

Furthermore, the continuous involvement of stakeholders enriched the criteria definition and the interpretation of results, grounding the analysis in real‐world clinical and experiential knowledge. Inputs gathered from stakeholders during the initial workshop were crucial for criteria identification and definition, ensuring that they were clinically relevant and meaningful in real‐world settings. For example, the discussion highlighted the importance of considering treatment discontinuation for AEs as a proxy for safety, to better reflect the toxicity burden of the therapy, and the inclusion of QoL for providing a comprehensive assessment of the drug's profile. Moreover, the second workshop offered valuable insights that helped contextualize and interpret the results. Stakeholders unanimously underlined the critical importance of efficacy and safety in treatment decisions. However, efficacy was considered more relevant for the IGHV^unmut^ population due to its more aggressive disease course, shorter overall survival, and higher variability in drug performance [4, 6]. In contrast, for the IGHV^mut^ population, clinicians placed greater emphasis on treatment duration, reflecting the population's more favorable prognosis and narrower efficacy range. This preference aligns with ESMO guidelines, which recommend fixed‐duration regimens when efficacy is comparable [4]. Across both populations, patients consistently prioritized QoL more than other respondents, reflecting their lived experience with treatment side effects and day‐to‐day impact. In contrast, payers and methodologists assigned more weight to drug costs than other groups, due to their roles in reimbursement and budgetary decision‐making. These differences in preferences help explain the distinct treatment choices observed across stakeholders and IGHV subgroups. In the IGHV^mut^ population, clinicians favored venetoclax + ibrutinib, primarily due to its time‐limited regimen, while patients leaned toward zanubrutinib, mainly due to the importance attributed to QoL. In the IGHV^unmut^ population, where efficacy was paramount and treatment duration less influential, second‐generation BTKis, zanubrutinib and acalabrutinib, emerged as the top choices across all stakeholder groups.

While no previous MCDA has been conducted in the context of CLL in Italy, some studies using DCEs [5, 51, 52] have investigated treatment attribute prioritization. These studies explored important clinical and treatment‐related aspects such as efficacy, safety, mode of administration, treatment duration, and costs, criteria also considered in the present analysis. Notably, only one of these studies [51] included multiple stakeholder profiles (patients and clinicians). Our study builds on and complements these studies by incorporating additional dimensions, such as QoL, including the perspective of payers, and evaluating real‐world therapies. This approach allows for a more comprehensive and comparative value assessment. Moreover, the variability in stakeholder preferences observed in this study reflects a pattern reported in some MCDA studies in other onco‐hematologic settings in Italy [53, 54], where differences in prioritization emerged depending on the type of stakeholders involved. This finding further highlights the value of structured, multi‐stakeholder decision‐making approaches.

Although the study was conducted with methodological rigor and a novel approach, it also presents limitations. Firstly, at the time of analysis, no direct or indirect comparisons were available across all considered therapies. To address this, data were drawn from clinical trials and literature sources, selecting the most comparable evidence available. Moreover, sensitivity analyses were conducted to test the robustness of results where uncertainty was greatest. Secondly, the sample size only allowed for descriptive analyses and did not support inferential statistics. However, the sample size was larger than the median for MCDA studies [55], and inferential analyses are neither commonly applied in MCDA settings [55] nor required by the MACBETH methodology [24, 34]. Furthermore, while stakeholder representativeness was carefully ensured for clinicians, payers, and methodologists, the anonymity of patient participants prevented the collection of socio‐demographic or clinical data, making it impossible to assess whether they were representative of the target population.

Despite these limitations, this study represents a robust, methodologically sound, and innovative contribution to the field, offering valuable insights into stakeholder‐informed decision‐making in CLL treatment.

## 5 Conclusions

This study is the first MCDA focused on first‐line treatment selection for CLL, revealing that stakeholder preferences in Italy vary by role and IGHV mutational status. For the IGHV^mut^ population, venetoclax + ibrutinib and zanubrutinib were the most valued options, while in the IGHV^unmut^ population, second‐generation BTKis, zanubrutinib and acalabrutinib, were the preferred therapies. These findings highlight MCDA's value in supporting nuanced, stakeholder‐informed decisions that complement clinical guidelines and economic evaluations.

## Author Contributions

All authors contributed to the conception and design of the study, interpreted the data, and read and approved the final manuscript. A.D.C., F.F., R.M., and C.V. collected and analyzed the data.

## Funding

This study was sponsored by BeOne Medicines Ltd.

## Ethics Statement

This study was conducted in accordance with the Declaration of Helsinki. The questionnaire was preceded by an introductory page describing the study objectives and a subsequent informed consent page detailing key aspects of participation, including guarantees of anonymity. Participants were able to proceed to the questionnaire only after providing their consent to participate. This study consisted of an anonymous online survey designed to gather patients', clinicians', payers', and methodologists' views and preferences. It did not involve any intervention, product testing, or impact on patient care. The survey did not influence clinical management, behaviors, or medical follow‐up. No personally identifiable data were collected. Participation was entirely voluntary, and respondents could withdraw at any stage if they preferred not to continue.

## Consent

All participants provided written informed consent.

## Conflicts of Interest

P.S. has received research funding from Novartis; consulting fees from AbbVie, AstraZeneca, BeOne Medicines Ltd, and Johnson & Johnson; honoraria from AbbVie, AstraZeneca, BeOne Medicines Ltd, and Johnson & Johnson; and received a fee for participation in this study. F.B. has received consulting fees from IQVIA Solutions Italy Srl. C.C. is an employee of, had a leadership role in, and owns stock with BeOne Medicines Ltd. At the time of project initiation, A.D.C., F.F., R.M., and C.V. were employees of IQVIA Solutions Italy Srl, which receives professional service fees for a large number of activities in biopharma from a large number of sponsors, including BeOne Medicines Ltd. S.M. is an employee of and owns stock with BeOne Medicines Ltd. A.C. has received travel support from AbbVie, AstraZeneca, BeOne Medicines Ltd, Janssen, and Lilly; honoraria from AbbVie, AstraZeneca, BeOne Medicines Ltd, Janssen, and Lilly; consulting fees from AbbVie, AstraZeneca, BeOne Medicines Ltd, Janssen, and Lilly; and received a fee for participation in this study. A.M. and M.R. received a fee for their participation in this study.

## Permission to Reproduce Material From Other Sources

The authors have nothing to report.

## Data Availability

The data that support the findings of this study are available on request from the corresponding author. The data are not publicly available due to privacy or ethical restrictions.

## MACBETH Method

### Weighting

The section was designed to assess the relative importance of each criterion. Participants were shown potential improvements related to each criterion and asked to identify the one they considered most important. They rated its importance using six semantic categories ranging from “very weakly important” to “extremely important”. After selecting the top improvement, they were then asked to choose the second most important one and compare it to the first. This pairwise comparison process was repeated for all criteria improvements and was conducted twice: once for the mutated immunoglobulin heavy‐chain variable region (IGHV) gene (IGHV^mut^) population and once for the unmutated IGHV (IGHV^unmut^) population. Through this iterative comparison, participants implicitly distributed weights across all criteria, reflecting their perceived importance. Conceptually, each participant allocated a total of 100% importance across the criteria, assigning higher percentages to those considered more critical.

### Scoring

The section was designed to assess stakeholders' preferences for changes in performance within each criterion with more than two levels. Respondents were asked to express their preferences through pairwise comparisons between best and worst, intermediate and worst, and best and intermediate reference values in each criterion. In each comparison, they selected from six statements ranging from “very weakly preferable*”* to “extremely preferable.” In cases where a criterion was included in both the IGHV^mut^ and the IGHV^unmut^ populations but exhibited different performance levels across them, the corresponding scoring tasks were administered independently for each population. The scores attributed to each criterion were used to construct individual value functions. These functions assigned a score of 100 points to the best performance level and 0 points to the worst, while the intermediate level received a score determined by the relative strength of preference expressed in the comparisons. Depending on how participants valued the differences between levels, the resulting value functions could be convex (if the preference between best and intermediate was stronger), concave (if the preference between intermediate and worst was stronger), or linear (if both comparisons were valued equally).

### Aggregate Scores

For both IGHV^mut^ and IGHV^unmut^ populations, aggregate scores for the considered therapeutic alternatives were calculated for each participant using an additive aggregation approach. Following methodologies established in previous research [24, 34], the aggregate score *S*_*j*_(*a*) for a given alternative *a* and participant *j* was determined by the formula:

$$S_{j}\left(a\right)=\sum_{i=1}^{c}w_{j,i}v_{j,i}\left(a\right)$$

In this expression, *v*_*j,i*_(*a*) represents the value score assigned to the performance of alternative *a* based on participant *j'*s value function for criterion *i*, while *w*_*j,i*_ denotes the weight that participant *j* attributed to that criterion. The variable *c* is the total number of criteria.

Based on the individual aggregate scores, the mean score was calculated for each stakeholder group. To ensure equal representation, the overall aggregate score was then computed as the average of these group‐level means, thereby assigning equal weight to each stakeholder group regardless of its size.
